# Semiconductor NbSe_2_ nanoparticles synthesized at various temperatures: a novel promising antifungal candidate with *in vitro* wound healing potential

**DOI:** 10.1039/d5na00116a

**Published:** 2025-08-11

**Authors:** Shivani R. Bharucha, Mehul S. Dave, Sunil H. Chaki, Tushar A. Limbani, Ashish Bhatt, Apurva C. Kadia

**Affiliations:** a N. V. Patel College of Pure & Applied Science, Charutar Vidya Mandal University Gujarat 388120 India mehul@nvpas.edu.in; b P. G. Department of Physics, Sardar Patel University Gujarat 388120 India; c C. L. Patel Institute of Studies and Research in Renewable Energy, New Vallabh Vidyanagar, Charutar Vidya Mandal University Gujarat 388121 India tusharlimbani97@gmail.com; d Department of Microbiology, Shri Alpesh N. Patel Post Graduate Institute of Science & Research Anand 388001 India

## Abstract

This study reports the synthesis of niobium diselenide (NbSe_2_) nanoparticles at room temperature (RT), 70 °C, and 100 °C *via* a sonochemical method, followed by an assessment of their structural, morphological, antimicrobial, cytotoxic, and wound healing properties. X-ray diffraction, scanning electron microscopy, and transmission electron microscopy analyses revealed changes in the crystallinity, size, and morphology of the nanoparticles as a function of synthesis temperature. The sample synthesized at 70 °C exhibited the highest cytotoxicity against HaCaT cells, while those synthesized at RT and 100 °C showed moderate and low cytotoxicity, respectively. Antimicrobial properties varied: the RT nanoparticles showed the highest inhibition against *Aspergillus niger*, while the 100 °C nanoparticles demonstrated some inhibition against *Candida albicans*. Wound healing assays revealed that the RT synthesized nanoparticles promoted the highest wound closure (94.78%) at a concentration of 0.5 μg mL^−1^. These results highlight the influence of synthesis temperature on the biological properties of NbSe_2_ nanoparticles, suggesting their potential for therapeutic applications in regenerative medicine, with the RT sample showing the most promising outcomes.

## Abstract 2

NbSe_2_ nanoparticles synthesized at RT, 70 °C, and 100 °C *via* sonochemical method show temperature-dependent bioactivity. RT sample exhibits best wound healing (94.78%) with minimal cytotoxicity, highlighting potential for regenerative medicine.

## Graphical abstract

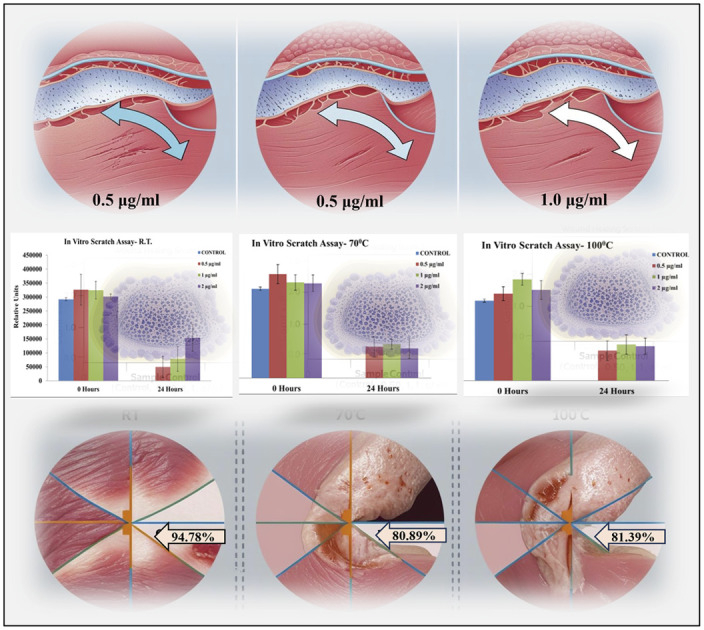

## 1. Introduction

Bacterial infections remain a critical global public health issue, affecting 17 billion people annually.^1^ Methicillin-resistant *Staphylococcus aureus* (MRSA) and other drug-resistant bacteria have emerged as a result of the excessive and inappropriate use of antibiotics, posing a severe challenge to conventional therapies.^2^ As bacterial resistance rises, alternative therapeutic strategies are needed.^3^ While techniques such as ultraviolet disinfection and chemical decontamination have been explored, nanotechnology, which uses metal and semiconductor nanoparticles, offers promising new solutions.^4^

Niobium diselenide (NbSe_2_), a transition metal dichalcogenide (TMD), is gaining recognition for its potential biomedical applications, particularly in wound healing.^5^ As a semiconductor nanoparticle, NbSe_2_ offers advantages such as tunable electronic and optical properties, alarge surface area, and biocompatibility, making it an effective antibacterial agent.^6^ Its mechanisms of action include reactive oxygen species (ROS) generation, membrane disruption, and biofilm inhibition, which are the key factors in treating bacterial infections.^7^

While TMDs such as MoS_2_ and WS_2_ have been explored in biological contexts, NbSe_2_ has been primarily studied for electronic and superconducting applications.^8^ However, its semiconducting properties, combined with a layered structure, enable efficient light absorption and heat generation, making it highly suitable for antibacterial therapies.^9^ Under near-infrared (NIR) radiation, NbSe_2_ nanoparticles generate localized heat through photo-thermal effects, leading to bacterial inactivation and biofilm disruption, which are crucial for effective wound healing.^10^ NbSe_2_'s biocompatibility further ensures its safety in biological environments, with minimal toxicity. Recent studies on similar TMDs, such as NbS_2_, have demonstrated their effectiveness in wound healing.^11,12^ These nanoparticles exhibit enhanced photo-thermal conversion efficiency under NIR irradiation, promoting the rapid thermal killing of Gram-positive and Gram-negative bacteria while inhibiting biofilm formation, thus reducing the risk of drug resistance.^13,14^ This points to the potential of NbSe_2_ nanoparticles to perform similarly, offering a dual function in wound healing: eradicating pathogens and supporting tissue regeneration.^15^

The biological reduction of metallic ions or oxides into nanoparticles under ambient conditions is a rapid, scalable process. Metal ions are known to interact with biomolecules, and their nanoparticle preparation can further enhance their bioactive properties such as antibacterial, antifungal, antioxidant, and anticancer activities.^16^ The rise of multidrug-resistant microorganisms, driven by antibiotic misuse, poses a critical threat to human health. Developing novel antibiotics with unique mechanisms is essential. Metals and their oxides exhibit potent antimicrobial activities at low concentrations.^17^ Additionally, increasing infections in plants and animals are a global concern. Metal nanoparticles are known to show antimicrobial activities against bacteria and fungi. The broad-spectrum bioactivity of nanoparticles makes them promising antimicrobial agents for both agricultural and medical applications.^18^ Nanoparticles can function as antioxidants and counteract oxidants by neutralizing free radicals. Antioxidant molecules achieve this by donating electrons to free radicals, thereby mitigating cellular damage.^19^ Numerous studies have demonstrated that metal nanoparticles (NPs) are biocompatible and non-toxic to normal human cells. Additionally, their ability to localize in specific areas enables targeted drug delivery. Metal NPs also exhibit significant anticancer, antimicrobial, and antiviral properties.^20^

Despite their promising short-term safety profile, comprehensive biological assessments, including long-term and dose-dependent toxicity studies, are essential to fully establish the clinical safety of NbSe_2_ nanoparticles. These would involve chronic exposure models and the evaluation of oxidative stress markers, apoptotic signalling, and genetic damage, among other parameters.

Furthermore, NbSe_2_ nanoparticles show potential for multimodal imaging applications.^21,22^ Their strong NIR absorption supports photothermal and photoacoustic imaging, and their layered structure enables functionalization with imaging dyes or contrast agents. While not intrinsically magnetic, NbSe_2_ can be engineered for magnetic resonance imaging (MRI) by doping with paramagnetic ions or integrating magnetic nanoparticles. This opens avenues for real-time, imaging-guided therapies combining diagnosis and treatment in a single platform.

In this study, the authors first report the effect of the synthesis temperature on the properties of these nanoparticles. By meticulously examining its influence on the structural, electronic, and antimicrobial characteristics of the nanoparticles, the authors aimed to gain deeper insights into how the synthesis temperature governed their properties and functionality. Through systematic characterization and performance evaluation, the authors also aimed to report, for the first time, the antimicrobial activity, including antibacterial, antifungal, antioxidant, and *in vitro* wound healing assay, of these NbSe_2_ nanoparticles. This comprehensive exploration sheds light on their potential biomedical applications and paves the way for further advancements in the field.

## 2. Materials and methods

### 2.1. Synthesis of NbSe_2_ nanoparticles influenced by the synthesis temperature

**Fig. 1:**
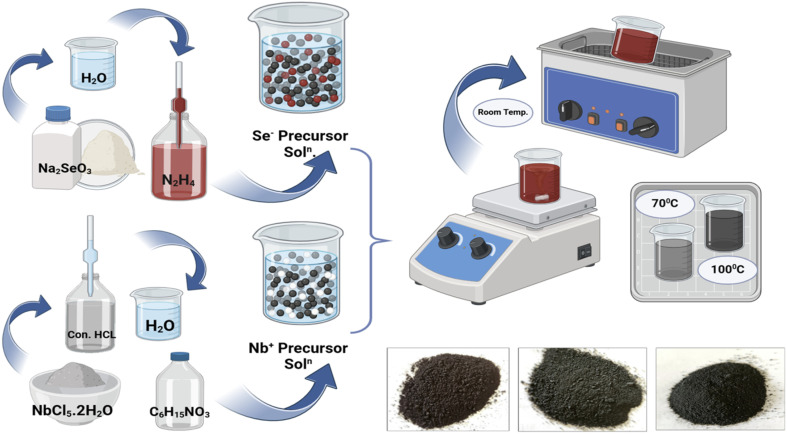
The schematic of the synthesis process.

The synthesis of NbSe_2_ nanoparticles, as previously reported^23,24^ and as shown in Fig. 1, involved preparing a solution of 1 M NbCl_2_·2H_2_O in 5 mL of 50% diluted HCl, followed by ultrasonic treatment for 15 minutes. TEA (2.79 mL) was added to the solution, and a mixture of Na_2_SeO_4_ and hydrazine hydrate, prepared in deionized water, was introduced drop-wise under continuous ultrasonic treatment, with the total volume adjusted to 50 mL. The solution underwent an additional hour of sonochemical treatment, forming a dark brown suspension. The precipitates were filtered, washed, and dried at ambient temperature for 8 hours to yield NbSe_2_ nanoparticles. This method was adapted in our study to include synthesis at the elevated temperatures of 70 °C and 100 °C, and all nanoparticles were subsequently analyzed for their thermal properties to investigate the effects of the synthesis temperature.^23^

### 2.2. Antimicrobial activity

NbSe_2_ nanoparticles were assessed for their antimicrobial potential against bacteria as well as fungi. The antimicrobial activity was assessed by the zone of inhibition method (Kirby–Bauer assay). Bacterial cultures were grown in a nutrient broth till their optical density reached 0.5, which is as per McFarland standards (approximately 1.5 × 10^8^ CFU mL^−1^), and 100 μL was spread onto Mueller–Hinton agar (MHA) plates using a sterile spreader. Discs containing 10 μL of various concentrations ranging from 0 to 1000 μg per disc were placed on the agar in a dose-dependent manner. A disc containing only the solvent served as the vehicle control, while a disc with 10 μg of ciprofloxacin was used as the positive control. The plates inoculated with *Escherichia coli* and *P. acnes* were incubated at 37 °C for 24 hours. The zones of inhibition around the discs were measured and documented. For antifungal activity, potato dextrose agar (PDA) plates for *Aspergillus niger* and Sabouraud Dextrose Agar (SDA) plates for *Candida albicans* were prepared. Solvent-loaded discs served as the vehicle controls, while discs containing 50 μg of Amphotericin B served as the positive controls. The *A. niger* plates were incubated at 37 °C for 48 hours, and the *C. albicans* plates were incubated at 37 °C for 24 hours. The zones of inhibition around the discs were measured and recorded.

### 2.3. Antioxidant activity

**Fig. 2:**
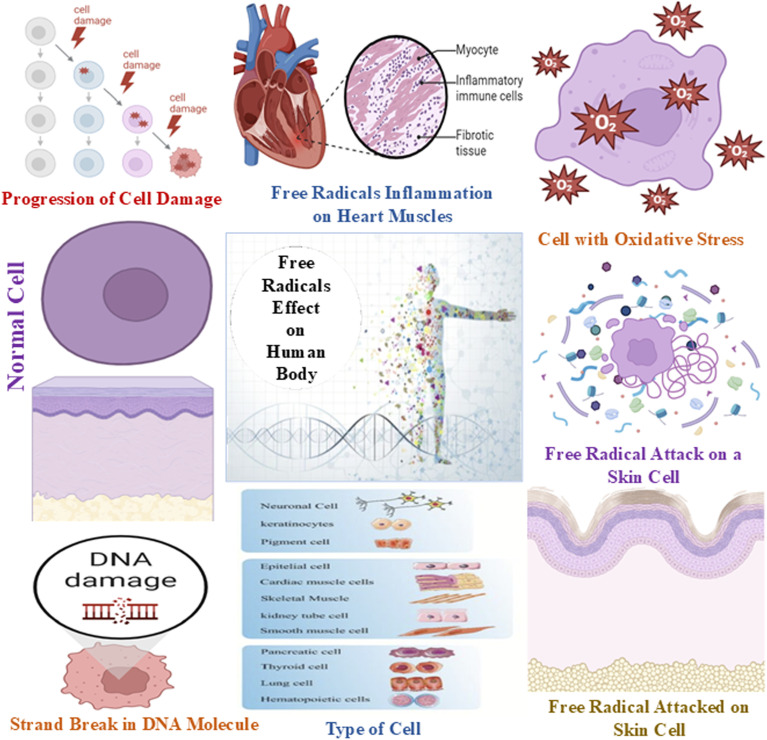
Schematic presentation of normal cells to radicals attacked and oxidative stress cells.

The existence of free radicals significantly impacts human health (Fig. 2), leading to various illnesses.^25^ Free radicals are recognized for their significant involvement in aging and inflammation. Consequently, biopolymers with antioxidant capabilities have been a focal point for several researchers in recent times.^26^ Numerous research studies have shown that the antioxidant properties of exopolysaccharides (EPSs) are linked to their chemical compositions and structural factors.^27^ The antioxidative properties are ascribed to several mechanisms, including the degradation of lipid peroxides, inhibition of chain reaction initiation, prevention of ongoing hydrogen abstraction, chelation of transition metal catalysis, reductive capacities, and free radical scavenging ability.^28^

#### 2.3.1. Ferric reducing antioxidant power (FRAP) assay

The FRAP assay, as described by Benzie and Strain (1996), is a simple, rapid, and highly reproducible method for assessing antioxidant activity. To perform the assay, 0.04 mL of 0.2 M sodium phosphate buffer (pH 6.6) and 0.05 mL of a 1% potassium ferricyanide [K_3_Fe(CN)_6_] solution were mixed with 10 μL of test samples at varying concentrations (0–1000 μg mL^−1^) or the standard ascorbic acid (0–50 μg mL^−1^). The reaction mixture was vortexed thoroughly and incubated at 50 °C for 20 minutes. Following incubation, 0.5 mL of 10% trichloroacetic acid (TCA) was added to terminate the reaction. Subsequently, 50 μL of a 0.1% ferric chloride solution and 50 μL of deionized water were added to the mixture. The absorbance of the resulting colored solution was measured at 750 nm using a microplate reader, with the blank serving as the reference.

#### 2.3.2. DPPH radical scavenging activity

The DPPH assay is highly sensitive and effective for detecting free radical scavenging activity in natural products and assessing their hydrogen-donating potential, even at low concentrations. In a 96-well plate, 0.1 mL of a 0.1 mM DPPH solution was mixed with 5 μL of test samples at varying concentrations (0–1000 μg mL^−1^). The reaction was performed in triplicate, with blank duplicates containing 0.2 mL of DMSO/methanol and 5 μL of the test samples (0–50 μg mL^−1^). The plate was incubated in the dark for 30 minutes. Decolorization was measured at 495 nm using a microplate reader. The control contained 20 μL of deionized water. The scavenging activity was expressed as “% inhibition” relative to the control.

#### 2.3.3. Cupric ion reducing antioxidant capacity (CUPRAC) assay

The CUPric reducing antioxidant capacity (CUPRAC) assay quantifies the total antioxidant capacity of both hydrophilic and hydrophobic materials. In a 96-well plate, 10 μL of test samples, a standard (Trolox), or a blank at varying concentrations was added to designated wells, followed by 200 μL of the reagent mixture (10 mM CuCl_2_, 1.0 M ammonium acetate buffer at pH 7.0, and neocuproine in equal proportions). The reaction mixtures were incubated in the dark for 30 minutes. The control reaction contained 20 μL of deionized water instead of the sample or standard. The absorbance was measured at 490 nm using a microplate reader. The scavenging activity was expressed as “% inhibition” relative to the control.

#### 2.3.4. ABTS radical scavenging activity

To prepare the ABTS free radical reagent, a 2.45 mM solution of ammonium persulfate (APS) and a 7 mM solution of ABTS were mixed and diluted 100-fold to generate ABTS radicals. In a 96-well plate, 10 μL of varying concentrations of the standard (ascorbic acid, 0–50 μg mL^−1^) and test samples (0–1000 μg mL^−1^) were added to 200 μL of the ABTS free radical reagent. The mixture was incubated at room temperature in the dark for 10 minutes. The absorbance of the resulting decolorization was measured at 750 nm using a microplate reader. The results, including the negative control, were recorded.

#### 2.3.5. Hydroxyl free radical scavenging activity

In a 96-well plate, 66 μL of the reagent mixture (composed of 10 μL of EDTA (0.5 M), 24.14 mg of deoxyribose, 88 μL of FeCl_3_ (10 mg mL^−1^), 28 μL of H_2_O_2_ (6%), and water up to 33 mL) was added, followed by 10 μL of the test sample, 24 μL of phosphate buffer (50 mM, pH 7.4), and 10 μL of ascorbic acid. The mixture was incubated at 37 °C for 1 hour. Gallic acid was used as the standard. After incubation, each well received 50 μL of 10% TCA and 50 μL of 1% tertiary butyl alcohol (TBA), resulting in the formation of a pink chromogen. The absorbance was measured at 540 nm using a microplate reader.

#### 2.3.6. Superoxide anion radical scavenging activity

Superoxide anion (O^2−^) radical scavenging activity was measured with slight modifications based on the method described by Yang *et al.* (2013).^63^ Superoxide radicals were generated in 3 mL of 16 mM Tris–HCl buffer (pH 8.0) containing varying concentrations of FmEPS (200–1000 μg mL^−1^), 300 μM nitro blue tetrazolium (NBT), 120 μM phenazine methosulfate (PMS), and 936 μM β-nicotinamide adenine dinucleotide. Ascorbic acid was used as the positive control. The reaction between NBT and superoxide anion radicals was measured spectrophotometrically at 560 nm to assess scavenging activity.

#### 2.3.7. Reactive nitrogen oxide scavenging assay

A reaction mixture was prepared by adding 10 μL of either the blank or the sample (gallic acid) to 40 μL of distilled water and 50 μL of 10 mM sodium nitroprusside. The mixture was pre-incubated for 15 minutes in light at room temperature. After incubation, 100 μL of the Griess reagent was added to both the test and control wells, followed by further incubation for 5–10 minutes at room temperature to allow chromophore formation and stabilization. The absorbance was measured using a microplate reader at 540 and 660 nm.

### 2.4. Cytotoxicity assay of NbSe_2_ nanoparticles

The anti-cell proliferation activity of the NbSe_2_ nanoparticles was assessed using the MTT assay on the adult human skin cell line HaCaT (immortal keratinocyte cell line). A 96-well plate was seeded with 10 000 cells per well and incubated for 24 hours at 37 °C with 5% CO_2_ in Dulbecco's modified Eagle medium (DMEM) supplemented with 10% fetal bovine serum (FBS) and a 1% antibiotic solution. The cells were treated with varying concentrations of the test materials the following day. After 24 hours of incubation, the MTT solution was added, and the cells were further incubated for 2 hours. Upon completion, the cell layer was dissolved in 100 μL of dimethyl sulfoxide (DMSO), and the absorbance was measured at 540 and 660 nm using an ELISA plate reader. The culture supernatant was then collected. The IC_50_ value was determined using GraphPad Prism 6 software. Images were captured using an inverted microscope (Olympus EK2) and a 10 MP Aptima CMOS digital camera (AmScope).

### 2.5. Wound healing assay for NbSe_2_ nanoparticles

The wound healing assay was performed on HaCaT cells. The cell line and necessary chemicals and research facilities were provided by Aakaar Biotechnologies Private Limited, Uttar Pradesh, India. HaCaT cells (10 000 cells per well) were seeded in a 96-well plate and incubated at 37 °C in a humidified atmosphere with 5% CO_2_ for 24 hours. The cells were maintained in the DMEM medium supplemented with 10% fetal bovine serum (FBS) and a 1% antibiotic–antimycotic solution. After 24 hours, a scratch was introduced using a 200-μL pipette tip to create a uniform gap in the cell monolayer. Cells were then treated with varying concentrations of the test formulation. Images were captured at specific time points (0–24 hours) using an AmScope digital camera (10 MP Aptima CMOS) attached to an inverted microscope (Olympus EK2). The gap area was quantified using ImageJ software (NIH), and the results were graphically represented.

## 3. Results and discussion

### 3.1. Synthesis temperature influence on nanoparticles

**Fig. 3:**
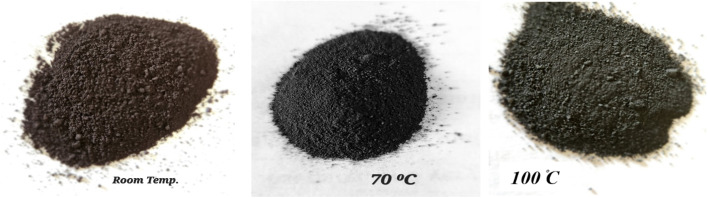
Photographs of NbSe_2_ nanoparticles synthesized at different temperatures.

Fig. 3 shows the synthesized NbSe_2_ nanoparticles at room temperature (RT), 70 °C, and 100 °C with distinct color changes, reflecting the material's complex properties and phase behavior. The synthesized nanoparticles exhibit notable color variations depending on the synthesis temperature. The RT synthesized nanoparticles display a distinct brown color, which remains consistent up to 70 °C, indicating minimal changes in their optical properties or surface characteristics within this range. However, above 70 °C, the color begins to change, transitioning from light black to dark black. This change becomes more pronounced as the temperature increases, culminating in a deep black hue at 100 °C. These variations in the color likely reflect changes in the particle size, morphology, or surface properties influenced by the elevated synthesis temperatures, suggesting a potential correlation between synthesis conditions and the optical or structural characteristics of the nanoparticles.^23^

### 3.2. Elemental analysis by EDAX

**Fig. 4:**
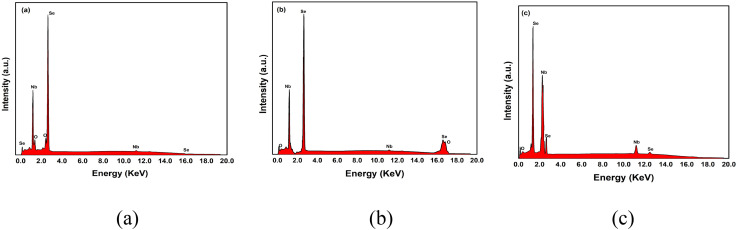
EDX spectra of NbSe_2_ nanoparticles synthesized at (a) RT, (b) 70 °C, and (c) 100 °C.

The elemental analysis of NbSe_2_ nanoparticles synthesized at RT, 70 °C, and 100 °C was conducted using energy-dispersive X-ray spectroscopy (EDX). The EDX spectra are shown in Fig. 4. This analytical technique provides valuable insights into the elemental composition of the synthesized nanoparticles.

The elemental compositions observed by EDX are tabulated in Table 1. The values state that the synthesized nanoparticles have a perfect stoichiometry. With a change in the synthesis temperature, the composition remains stoichiometric, thus confirming the formation of perfect NbSe_2_.

**Table 1:** Observed elemental percentages along with the standard values for NbSe_2_ nanoparticles synthesized at RT, 70 °C, and 100 °C

Sample	Elements							
	Nb				Se			
	wt%		at%		wt%		at%	
	Obs.	Stand.	Obs.	Stand.	Obs.	Stand.	Obs.	Stand.
RT	36.81	37.04	33.11	33.33	63.19	62.94	66.89	66.67
70 °C	37.96	37.04	33.31	33.33	62.96	62.94	66.69	66.67
100 °C	36.89	37.04	33.24	33.33	63.11	62.94	66.76	66.67

Thus, the composition study shows that despite variations in the synthesis temperature, the stoichiometry of NbSe_2_ remains perfect, indicating the robustness of the synthesis method across different thermal conditions. These findings propose that changes in the synthesis temperature do not alter the elemental composition of the synthesized nanoparticles.

### 3.3. Structural analysis

**Fig. 5:**
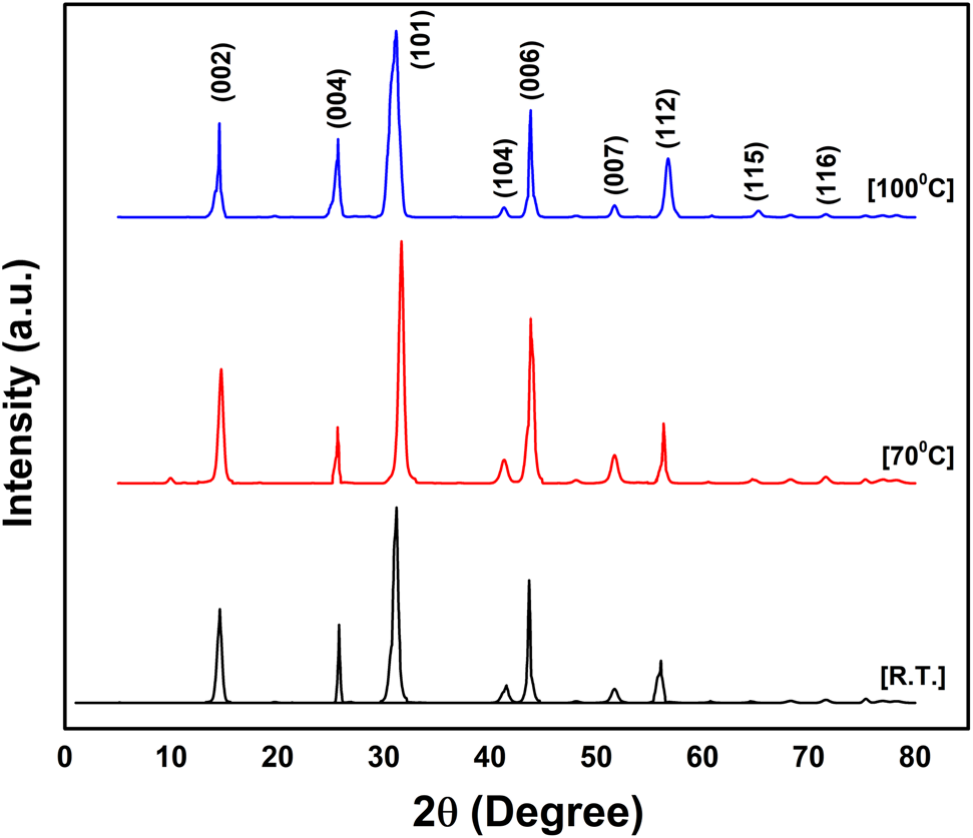
XRD profiles of sonochemically synthesized NbSe_2_ nanoparticles synthesized at RT, 70 °C, and 100 °C.

X-ray diffraction (XRD) analysis was performed to determine the crystalline structure and phase of NbSe_2_ nanoparticles synthesized *via* the sonochemical method. The results revealed a hexagonal crystal structure (space group *P*6_3_/*mmc*), consistent with the powder diffraction standard data (JCPDS).^29–31^ The diffraction patterns of samples synthesized at RT, 70 °C, and 100 °C, shown in Fig. 5, showed sharp, intense peaks, indicating high crystallinity. As the synthesis temperature increased, the intensity of the peaks became sharper, suggesting improved crystallinity.

The phase stability of NbSe_2_ was evident as no phase changes were observed across the different synthesis temperatures, highlighting the structural robustness of the material. The crystallite size was analyzed using Scherrer's equation:^32,33^1
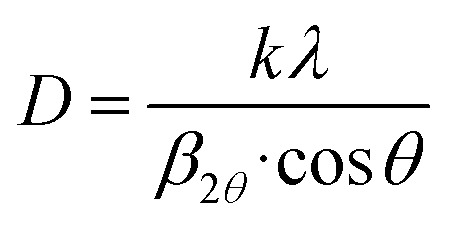
Here, *D* = the crystallite size, *k* = Scherrer constant (0.9) considering the crystallites to be spherical, *λ* = the wavelength, having a value of 1.5405 for the CuK_α_ radiation, *β* = the full-width half maximum, *θ* = Bragg's diffraction angle.

The calculated crystallite sizes were 14.94 Å, 15.12 Å, and 15.71 Å for the nanoparticles synthesized at RT, 70 °C, and 100 °C, respectively. Other structural parameters, such as dislocation density and internal strain, have been reported in a previous study.^23^ This slight increase in the crystallite size with temperature confirms the enhanced crystallinity of NbSe_2_ nanoparticles while maintaining their hexagonal phase stability across the investigated temperature range.

### 3.4. Morphological analysis

Scanning electron microscopy (SEM) and transmission electron microscopy (TEM) were employed to investigate the morphology of NbSe_2_ nanoparticles synthesized at different temperatures.

**Fig. 6:**
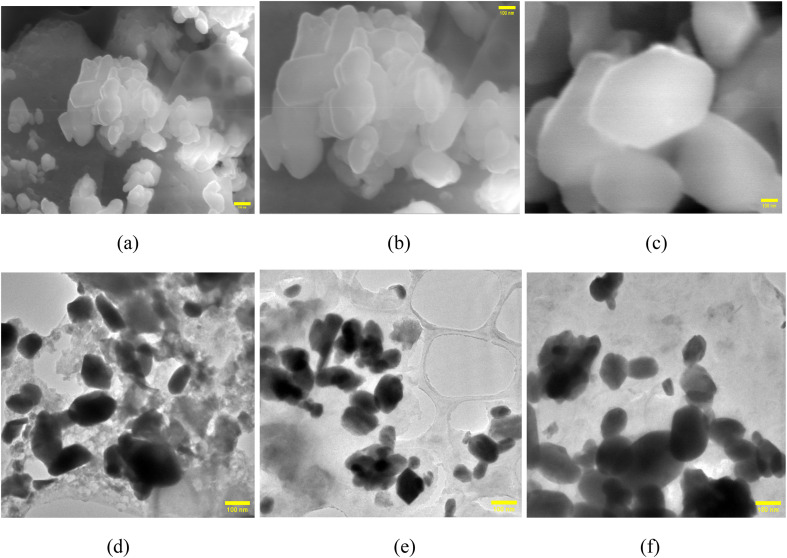
SEM (a–c) and TEM (d–f) images of sonochemically synthesized NbSe_2_ nanoparticles at (a and d) RT (b and e) 70 °C, and (c and f) 100 °C.

The SEM images of NbSe_2_ nanoparticles synthesized at RT, 70 °C, and 100 °C are shown in Fig. 6(a–c). The RT synthesized nanoparticles, Fig. 6(a), exhibit an aggregated and irregular morphology, with smaller crystallites forming clusters. As the temperature increases to 70 °C, Fig. 6(b), the particles become more uniform with improved crystallinity and reduced aggregation. This trend continues at 100 °C, Fig. 6(c), where the nanoparticles display a distinct hexagonal plate-like morphology with larger and well-defined edges.

The observed morphological changes are indicative of enhanced crystallinity at elevated temperatures, consistent with the increased thermal energy driving nucleation and growth processes. The reduced aggregation at higher temperatures suggests improved particle dispersion, which may be attributed to better reaction kinetics and surface stabilization during synthesis.^34^ These findings validate the temperature-dependent morphological evolution of NbSe_2_ nanoparticles and highlight the critical role of the synthesis temperature in tuning the material's structural characteristics.

The TEM images of NbSe_2_ nanoparticles synthesized at RT, 70 °C, and 100 °C are shown in Fig. 6(d–f). The TEM images clearly confirms the structural and morphological changes observed in the SEM analysis. The RT synthesized nanoparticles, Fig. 6(d), exhibit a relatively small size with noticeable agglomeration, likely due to weaker interparticle interactions. In the 70 °C synthesized nanoparticles, Fig. 6(e), the particles appear more dispersed with improved hexagonal shapes and slightly larger sizes, reflecting enhanced crystallization, whereas the 100 °C synthesized nanoparticles, Fig. 6(f), show a more uniform distribution and display distinct hexagonal platelets, corroborating the observations of SEM.

The increasing particle size with the temperature aligns with the Scherrer equation-based crystallite size calculations, which yielded crystallite sizes of 14.94 Å, 15.12 Å, and 15.71 Å for RT, 70 °C, and 100 °C, respectively. The TEM analysis provides direct evidence of temperature-induced improvements in particle uniformity and crystallinity, validating the role of the synthesis temperature in optimizing the properties of NbSe_2_ nanoparticles for potential applications.

### 3.5. Biological activity

#### 3.5.1. Antimicrobial potential of NbSe_2_ nanoparticles

##### 3.5.1.1. Antibacterial activity of NbSe_2_ nanoparticles

**Fig. 7:**
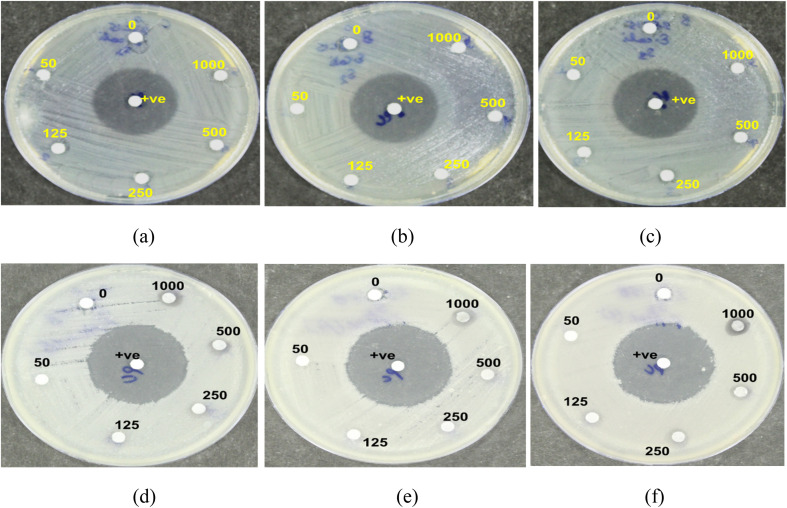
Antibacterial activities of (a and d) RT, (b and e) 70 °C, and (c and f) 100 °C NbSe_2_ nanoparticles against *E. coli* (a–c) and *P. acnes* (d–f).

This study investigated the antibacterial activity of NbSe_2_ nanoparticles synthesized at different temperatures of RT, 70 °C, and 100 °C against *Escherichia coli* and *Propionibacterium acnes*. The findings revealed that the nanoparticles did not exhibit significant antibacterial properties under the tested conditions. For *E. coli*, no visible zones of inhibition were observed, Fig. 7(a–c), for any of the samples, regardless of the concentration. This indicates that NbSe_2_ nanoparticles synthesized at RT, 70 °C, and 100 °C lack antibacterial activity against this organism. The positive control, ciprofloxacin, produced a pronounced zone of inhibition measuring 27.6 ± 0.57 mm. These results highlight the relatively weak or absent antibacterial properties of NbSe_2_ nanoparticles against *E. coli* under the tested conditions. The lack of activity can be attributed to factors such as the particle size or the intrinsic properties of NbSe_2_, which may limit its interactions with bacterial cell membranes or metabolic pathways compared to potent antibiotics like ciprofloxacin. Similarly, the NbSe_2_ nanoparticles demonstrated no significant antibacterial effects against *P. acnes*. A faint zone of inhibition, approximately 5.2 ± 0.57 mm in diameter, was observed, Fig. 7(d–f), only at the highest concentration of the test compound. Still, this effect was minimal compared to that of ciprofloxacin, which produced a clear inhibition zone of approximately 33.6 ± 0.57 mm.

The absence of antibacterial activity for both tested bacteria suggests that NbSe_2_ nanoparticles require further optimization to become effective antibacterial agents. Improving their antibacterial activities may involve modifying synthesis parameters, such as reducing the particle size, surface functionalization, or doping with other elements, to enhance their interactions with bacterial cells. Similar to the present study, Sarwar S. and Chakraborti S. (2016)^64^ reported insignificant antibacterial efficacy of the synthesized ZnO nanoparticles against *Vibrio cholerae*, *Shigella flexneri*, *Pseudomonas aeruginosa*, *Mycobacterium smegmatis*, *Proteus vulgaris*, and *Bacillus subtilis*. In contrast to the presented results, Sivakumar *et al.* (2017) found significant antibacterial activity against *Escherichia coli*, *Salmonella typhi*, *Vibrio cholera*, *Pseudomonas aeruginosa*, and *Proteus mirabilis* for silver nanoparticles.^35^ Brahmbhatt *et al.* (2023) also reported the antibacterial activity of Fe_3_O_4_ nanoparticles.^17^

##### 3.5.1.2. Antifungal activity of NbSe_2_ nanoparticles

**Fig. 8:**
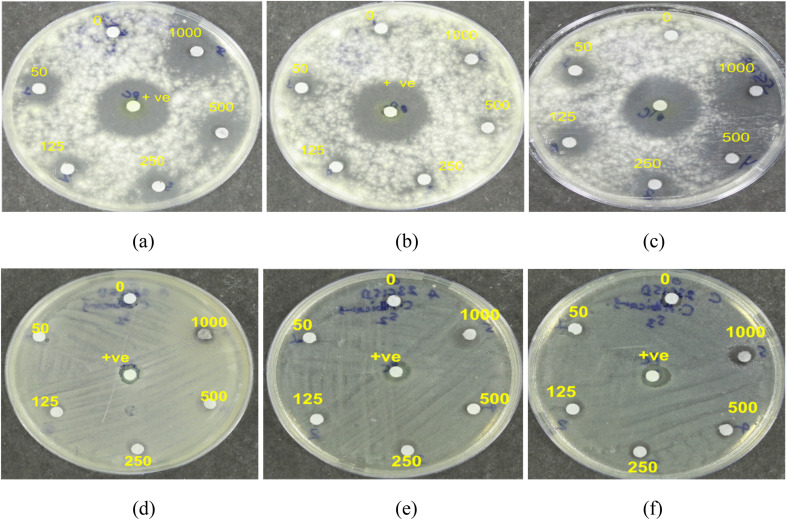
Antifungal activity of (a and d) RT, (b and e) 70 °C, and (c and f) 100 °C NbSe_2_ nanoparticles against *A. niger* (a–c) and *C. albicans* (d–f).

The synthesized samples showed variable antifungal activity against *Aspergillus niger* and *Candida albicans*. For *A. niger*, the sample prepared at RT exhibited the highest inhibition with a 20 mm zone at 1000 μg, followed by the sample synthesized at 100 °C, showing a 17 mm zone at 1000 μg, Fig. 8(a–c). The sample synthesized at 70 °C, Fig. 8(b), showed no antifungal activity. In contrast, Amphotericin B, the positive control, demonstrated superior potency with a 23 mm inhibition zone at just 50 μg. For *C. albicans*, only the sample synthesized at 100 °C, Fig. 8(f), displayed mild activity, with a 5 mm zone at 1000 μg, whereas those prepared at RT and 70 °C showed no inhibition. Amphotericin B again exhibited greater efficacy, with a 10 mm zone at 50 μg. The sample synthesized at RT showed the most promise against *A. niger*, while all samples were less effective than Amphotericin B. Following the COVID-19 pandemic, *Aspergillus niger* and *Candida albicans* have been identified as the major pathogens responsible for severe infections in immuno-compromised individuals, while healthy individuals typically remain unaffected. Of particular clinical relevance, *Candida albicans* has been linked to catheter-associated bloodstream infections, underscoring its significance in healthcare settings; thus, alternate antimicrobial agents like metal nanoparticles can have a huge impact in combating it.^36^ Similar to the present study, many reports on metal nanoparticles such as silver, nickel, magnesium, and copper have shown their great potential to become antifungal agents.^37^ Yadav *et al.* (2025) reported zinc–copper mixed metal oxide nanoparticles showing significant antifungal activity against *Alternaria alternata* and *Fusarium oxysporum*.^38^

##### 3.5.1.3. Proposed mechanism of the antimicrobial action of NbSe_2_ nanoparticles

**Fig. 9:**
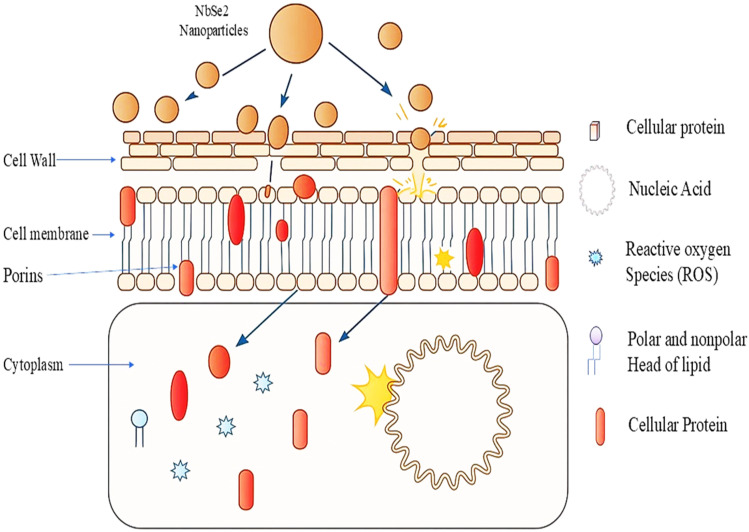
Proposed mechanism of the antimicrobial activity of NbSe_2_ nanoparticles.

There have been limited studies on the mechanism of the inhibition/lethal effects of metal nanoparticles on pathogens. It is known that metal nanoparticles can alter the surface chemistry, thus affecting the cell membrane structure of microorganisms.^39,40^ One of the under-reported mechanisms of metal nanoparticles is their ability to induce quantum confinement effects, where the restriction of the movement of ions may lead to altering the way biological macromolecules interact with each other, and it may often lead to cytotoxic effects.^41,42^Fig. 9 shows the proposed mechanism of the antimicrobial action of NbSe_2_ nanoparticles. NbSe_2_ nanoparticles are most likely involved in disrupting the cell wall and cell membrane due to their surface interaction capabilities. Once inside the cell membrane, they may interact with cellular proteins and nucleic acids, thus further having a negative effect on the metabolic activity of the cell. The metabolic and cellular function disruption can lead to the generation of reactive oxygen species (ROS) that can inhibit microbial growth. More studies are required to explore the mechanism by which metal nanoparticles interact with microorganisms. According to the current findings, the antimicrobial potential of nanoparticles can be further enhanced by optimizing their synthesis method and conditions.

#### 3.5.2. Antioxidant potential of NbSe_2_ nanoparticles

##### 3.5.2.1. FRAP assay

**Fig. 10:**
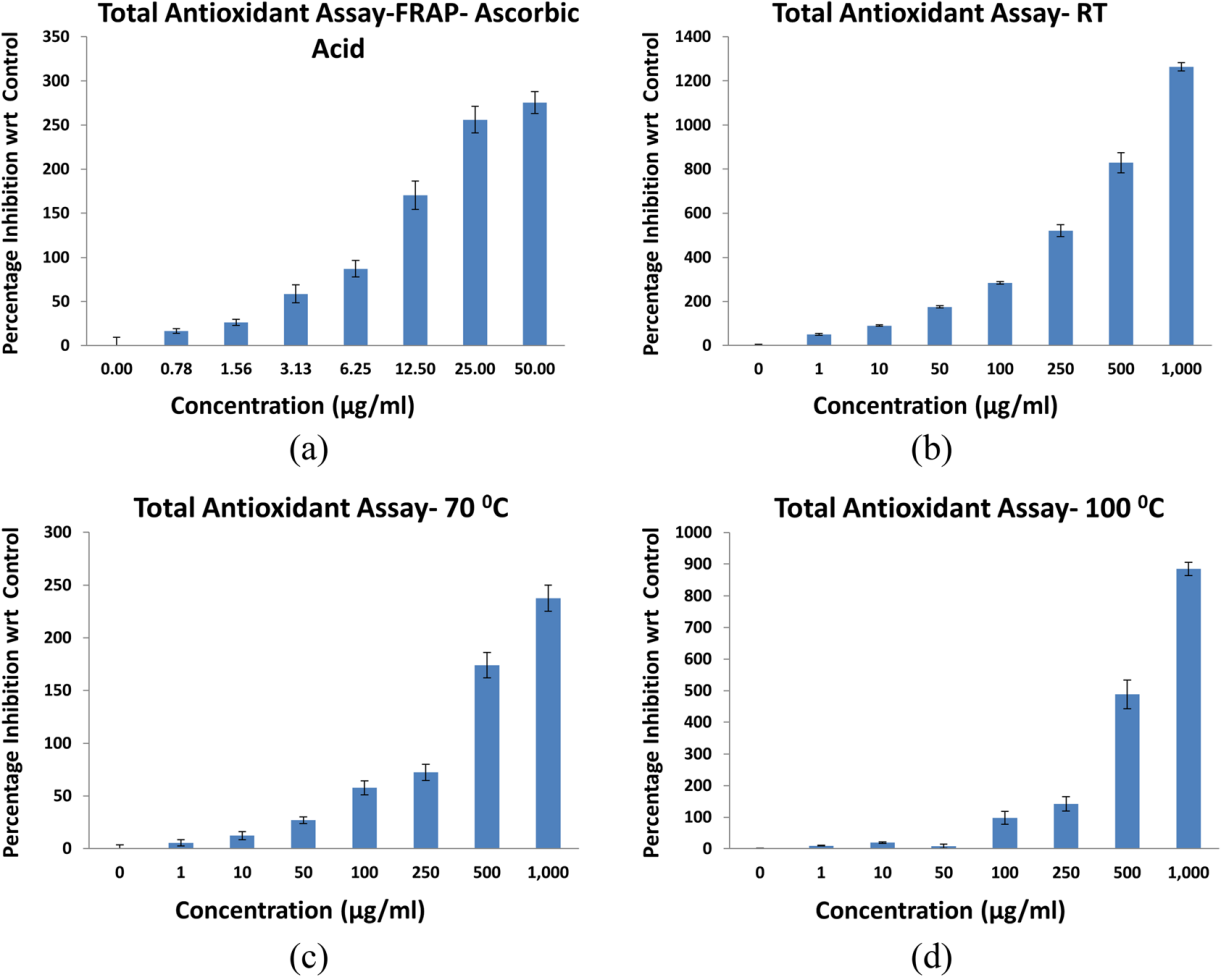
FRAP assays of (a) ascorbic acid and NbSe_2_ nanoparticles synthesized at (b) RT, (c) 70 °C, and (d) 100 °C.

The antioxidant activity of NbSe_2_ nanoparticles synthesized at RT, 70 °C, and 100 °C was assessed using the FRAP assay, with IC_50_ values indicating temperature-dependent variations in activity, Fig. 10.

The 70 °C synthesized nanoparticles exhibited the highest antioxidant potency, with IC_50_ = 93.85 ± 0.065 μg mL^−1^, followed by those synthesized at 100 °C showing IC_50_ = 65.19 ± 0.269 μg mL^−1^ and at RT showing IC_50_ = 0.9925 ± 1.241 μg mL^−1^. Ascorbic acid, used as a standard, showed an IC_50_ of 2.44 ± 0.23 μg mL^−1^. These results suggest that synthesis at 70 °C optimizes antioxidant activity, likely due to the improved particle size, morphology, and surface properties. The lower IC_50_ of the RT sample compared to that of ascorbic acid highlights its potential as a moderate antioxidant.

##### 3.5.2.2. DPPH radical scavenging activity

**Fig. 11:**
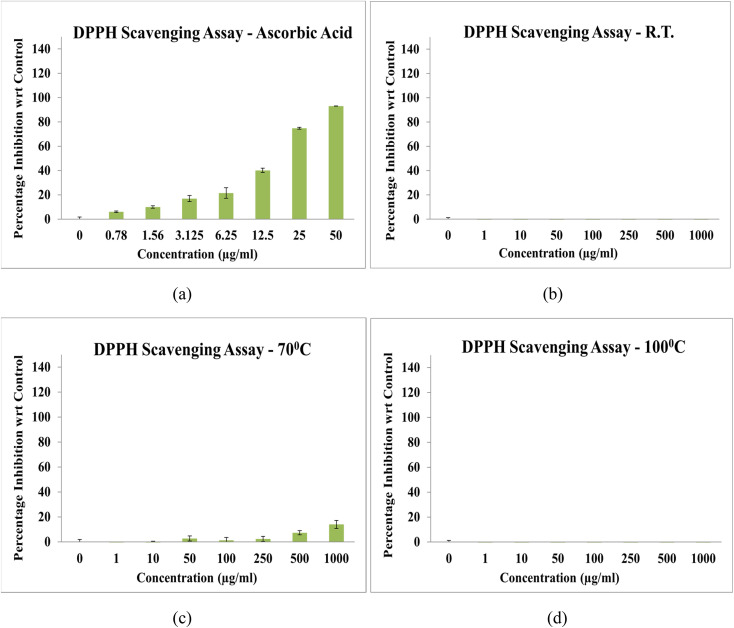
DPPH radical scavenging activity of (a) ascorbic acid and NbSe_2_ nanoparticles synthesized at (b) RT, (c) 70 °C, and (d) 100 °C.

The antioxidant activity of NbSe_2_ nanoparticles synthesized at RT, 70 °C, and 100 °C was evaluated using the DPPH free radical scavenging assay. All tested samples demonstrated minimal or no antioxidant activity compared to the standard antioxidant, ascorbic acid, with IC_50_ = 13.55 ± 0.23 μg mL^−1^, Fig. 11. The sample synthesized at 70 °C exhibited extremely low activity, with an IC_50_ exceeding 1000 μg mL^−1^, far beyond the assay's effective range. Nanoparticles synthesized at RT and 100 °C showed no measurable DPPH scavenging activity, indicating inactivity in this assay. These findings confirm that NbSe_2_ nanoparticles synthesized under these conditions lack significant DPPH radical scavenging properties. While the 70 °C sample displayed minimal activity, its efficacy remained negligible compared to ascorbic acid.

##### 3.5.2.3. CUPRAC assay

**Fig. 12:**
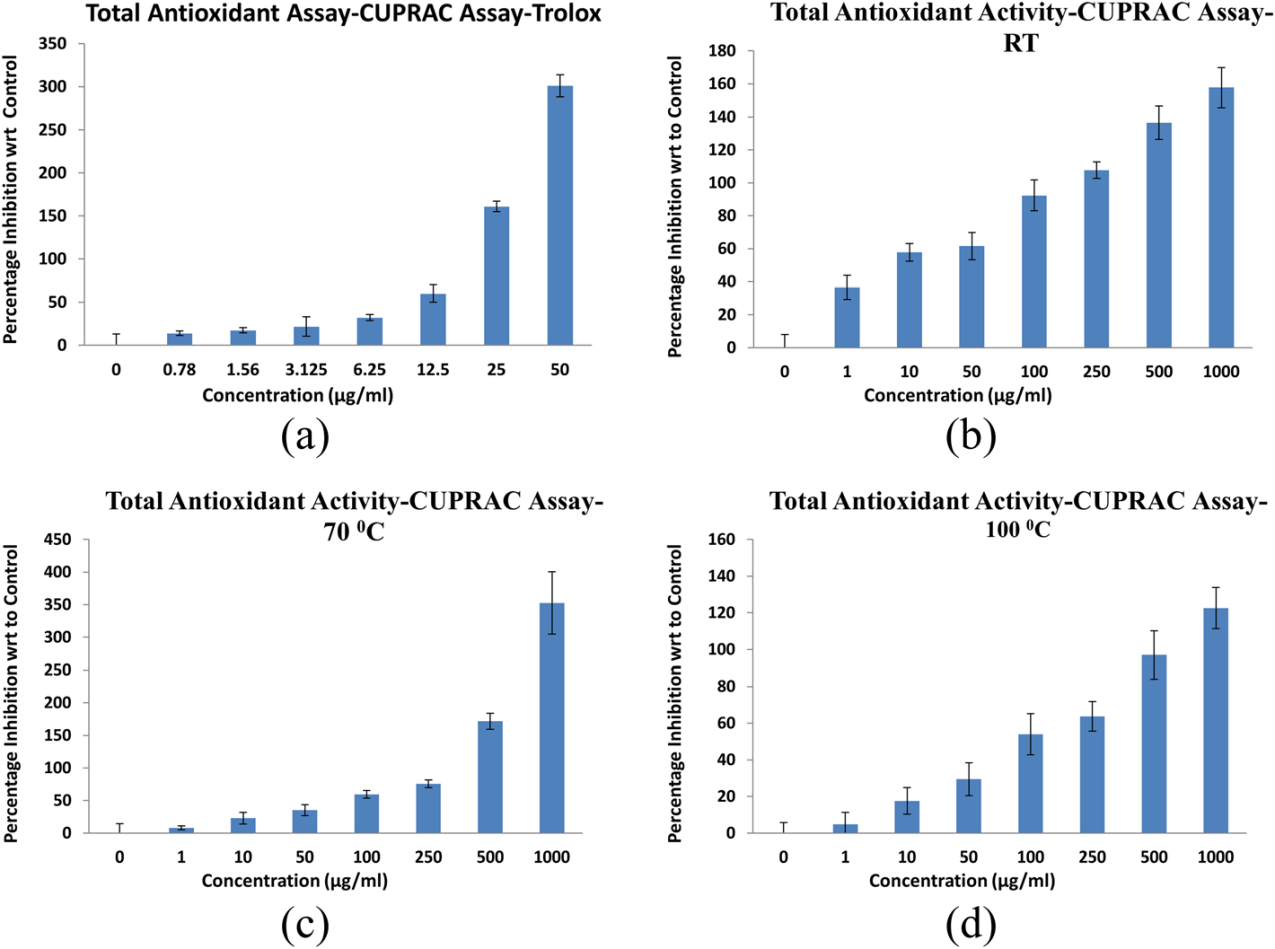
CUPRAC activity of (a) trolox, and NbSe_2_ nanoparticles synthesized at (b) RT, (c) 70 °C, and (d) 100 °C.

Fig. 12 shows the antioxidant activity of NbSe_2_ nanoparticles synthesized at RT, 70 °C, and 100 °C assessed using the CUPRAC assay. The IC_50_ values revealed a clear dependence of the antioxidant potential on the synthesis temperature, with the nanoparticles' effectiveness compared to the standard antioxidant, Trolox, showing IC_50_ = 8.02 ± 0.19 μg mL^−1^. The sample synthesized at RT exhibited the highest antioxidant activity with IC_50_ = 4.33 ± 0.63 μg mL^−1^, surpassing Trolox. The 70 °C sample showed moderate activity with IC_50_ = 72.63 ± 0.27 μg mL^−1^, while the 100 °C sample had the lowest activity with IC_50_ = 93.21 ± 0.13 μg mL^−1^, indicating diminished copper ion reducing capacity at elevated synthesis temperatures. Beğiç *et al.* (2021) reported similar findings, where they found significant CUPRAC activity for silver nanoparticles compared to various phenolic acids.^43^

These findings confirm that NbSe_2_ nanoparticles' antioxidant activity decreases with increasing synthesis temperature. The RT sample demonstrated superior antioxidant properties, with only 4.33 μg mL^−1^ to match the activity of 8.02 μg mL^−1^ of Trolox. This highlights its potential for applications demanding strong antioxidant activity. Conversely, the reduced efficacy of the 70 °C and 100 °C samples may limit their utility in such contexts.

##### 3.5.2.4. ABTS radical scavenging activity

**Fig. 13:**
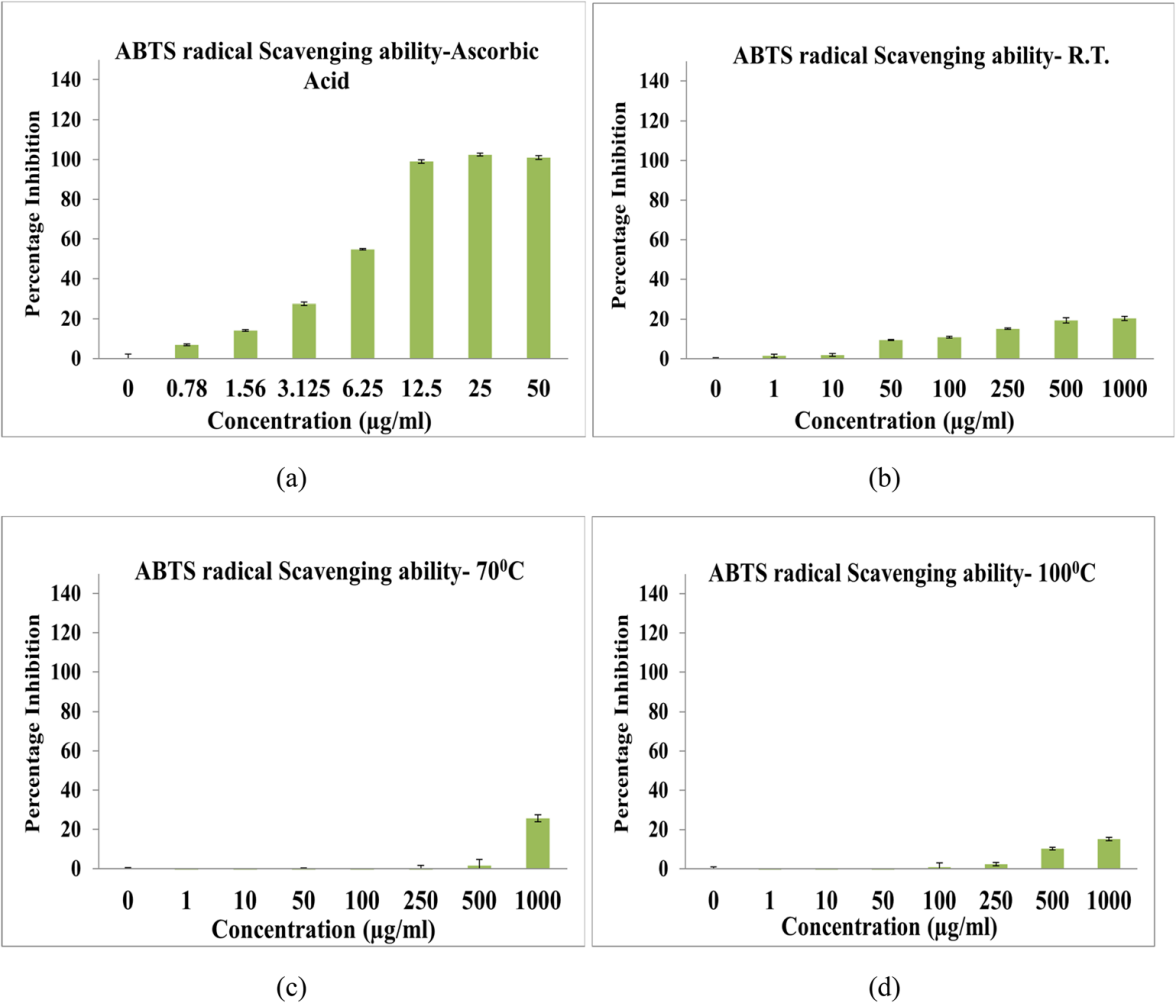
ABTS radical scavenging activity of (a) ascorbic acid and NbSe_2_ nanoparticles synthesized at (b) RT, (c) 70 °C, and (d) 100 °C.

The ABTS radical scavenging activity of NbSe_2_ nanoparticles synthesized at RT, 70 °C, and 100 °C was evaluated, revealing minimal antioxidant potential. All samples exhibited IC_50_ values exceeding the maximum tested concentration of 1000 μg mL^−1^, indicating insufficient activity to scavenge 50% of ABTS radicals even at the highest dosage, Fig. 13.

The RT sample showed no significant scavenging activity at 1000 μg mL^−1^, while the 70 °C and 100 °C samples similarly demonstrated negligible activity, with IC_50_ values also surpassing 1000 μg mL^−1^. In contrast, the standard antioxidant, ascorbic acid, displayed a robust radical scavenging ability with an IC_50_ of 4.92 ± 0.44 μg mL^−1^, underscoring the weak antioxidant capacity of the NbSe_2_ nanoparticles. The limited activity observed may stem from the intrinsic material properties, surface characteristics, or particle size, which likely reduce interactions with ABTS radicals. These findings indicate that NbSe_2_ nanoparticles, as synthesized, are unsuitable for applications requiring effective ABTS radical scavenging. To enhance their antioxidant capacity, modifications such as surface functionalization or particle size optimization should be explored.

##### 3.5.2.5. Hydroxyl free radical scavenging activity

**Fig. 14:**
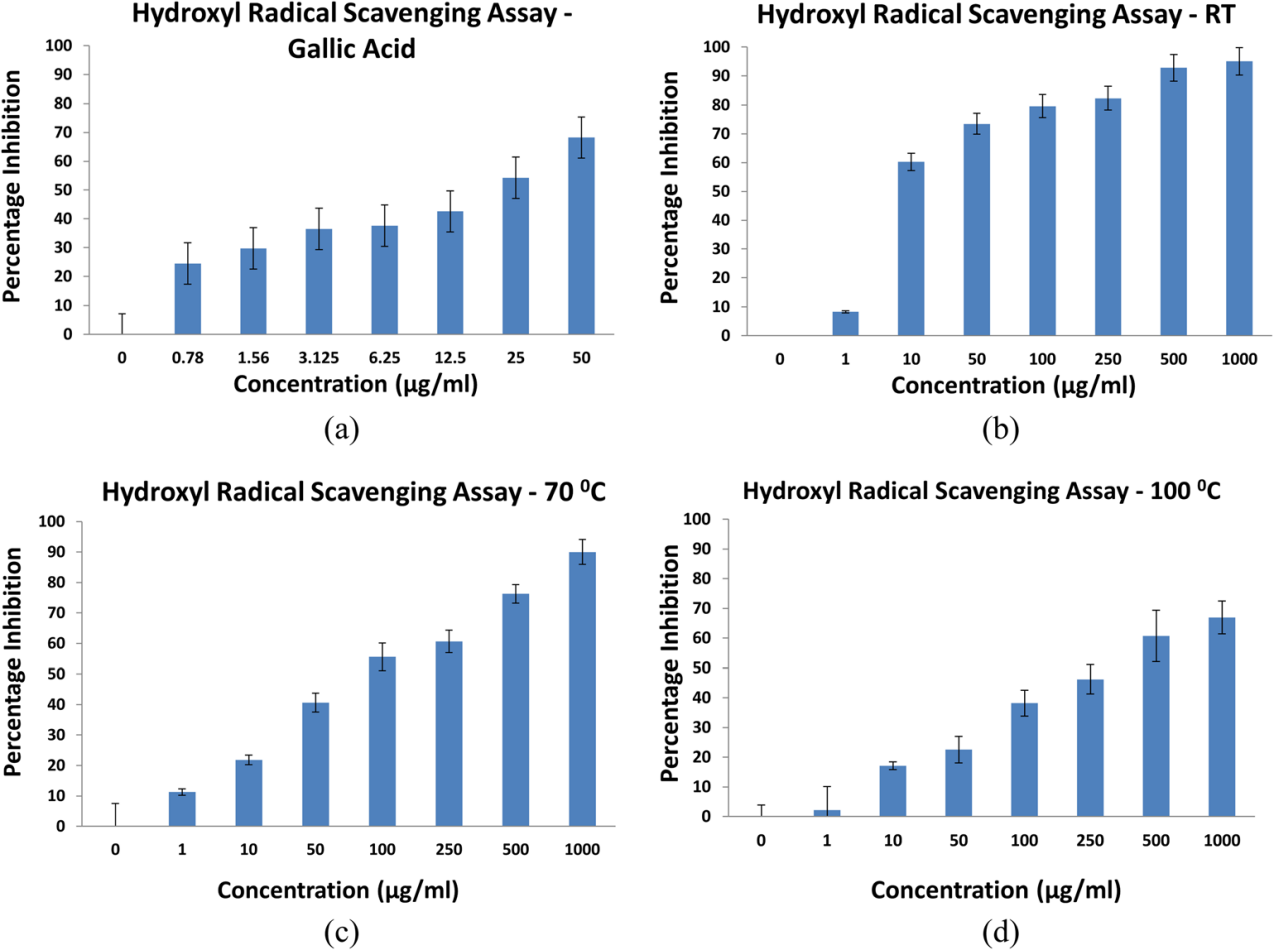
Hydroxyl radical scavenging activity of (a) gallic acid and NbSe_2_ nanoparticles synthesized at (b) RT, (c) 70 °C, and (d) 100 °C.

The hydroxyl radical scavenging activity of NbSe_2_ nanoparticles synthesized at RT, 70 °C, and 100 °C was evaluated using gallic acid as a reference. The IC_50_ values highlighted significant differences in the antioxidant potential among the samples. The sample synthesized at RT exhibited the strongest activity, with an IC_50_ of 10.08 ± 0.13 μg mL^−1^, outperforming gallic acid showing IC_50_ = 14.88 ± 0.09 μg mL^−1^, Fig. 14. This demonstrates the RT sample's superior ability to neutralize hydroxyl radicals. The 70 °C sample showed moderate activity with IC_50_ = 79.70 ± 0.07 μg mL^−1^, while the 100 °C sample exhibited the weakest scavenging potential with IC_50_ = 279.70 ± 0.05 μg mL^−1^, requiring significantly higher concentrations to achieve comparable radical inhibition.

##### 3.5.2.6. Superoxide anion radical scavenging activity

**Fig. 15:**
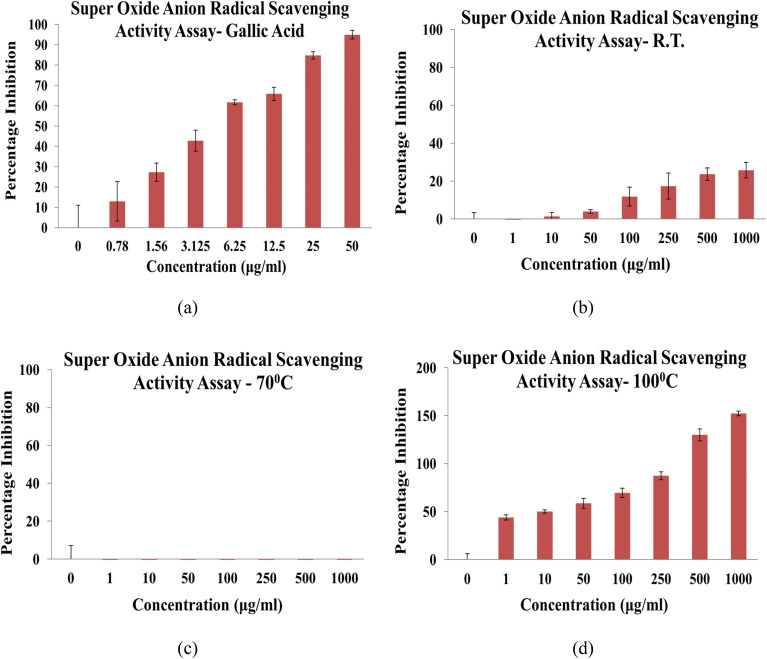
Superoxide anion radical scavenging activity of (a) gallic acid and NbSe_2_ nanoparticles synthesized at (b) RT, (c) 70 °C, and (d) 100 °C.

The superoxide anion scavenging activity of NbSe_2_ nanoparticles synthesized at RT, 70 °C, and 100 °C was evaluated and compared to that of gallic acid as the standard antioxidant. The results revealed the strong dependence of scavenging capacity on the synthesis temperature. The sample synthesized at 100 °C exhibited significant activity, with an IC_50_ of 5.48 ± 0.68 μg mL^−1^, comparable to gallic acid, with IC_50_ = 4.47 ± 0.04 μg mL^−1^, Fig. 15. This indicates the 100 °C sample's high efficiency in neutralizing superoxide radicals, requiring only slightly higher concentrations than the standard. In contrast, the RT sample displayed minimal activity, insufficient for matching either the 100 °C sample or gallic acid. The sample synthesized at 70 °C was largely inactive, showing no significant scavenging of superoxide radicals. These findings highlight the critical role of the synthesis temperature in determining the antioxidant properties of NbSe_2_ nanoparticles. Fig. 15 shows the results of the superoxide anion scavenging activity.

##### 3.5.2.7. Reactive nitrogen oxide scavenging assay

**Fig. 16:**
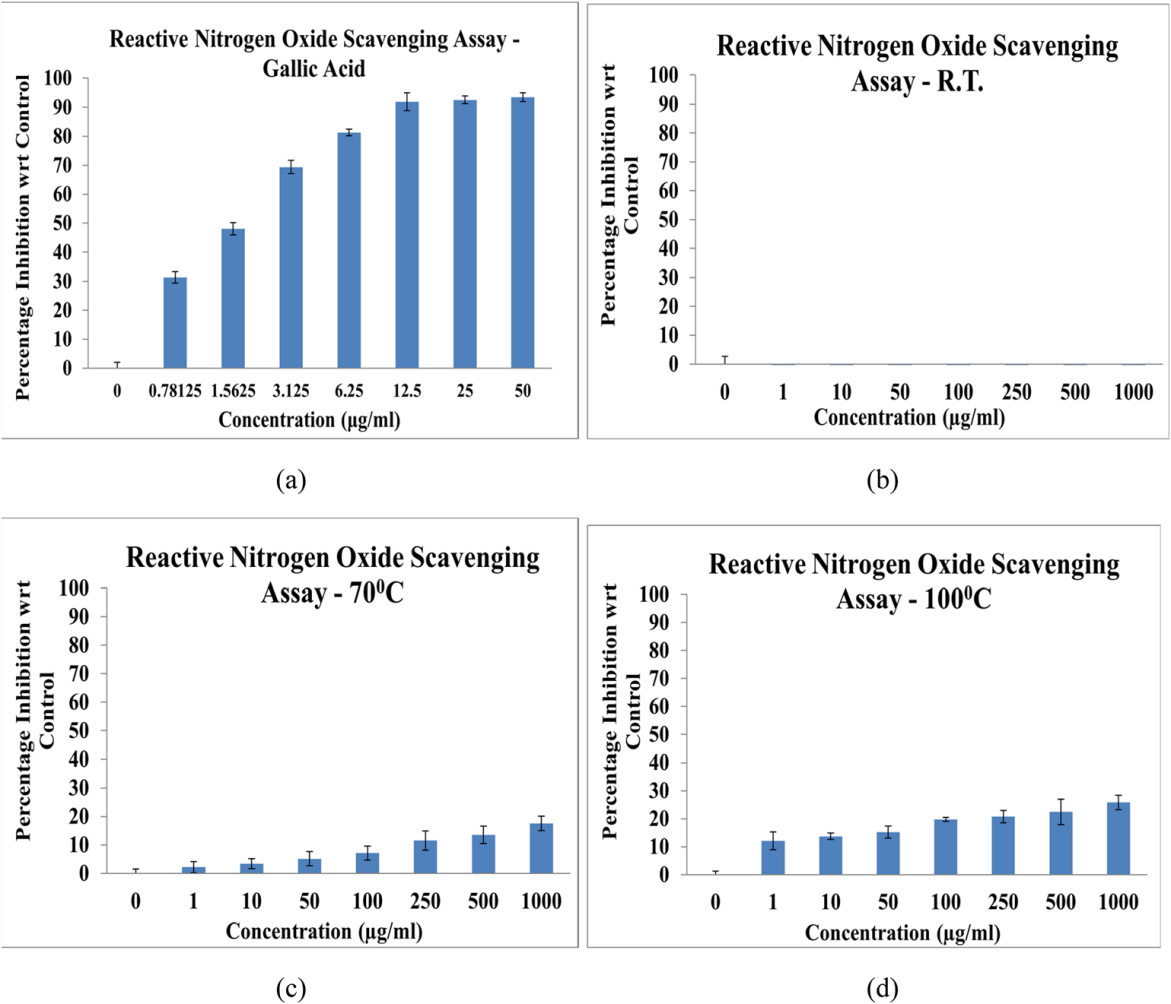
Reactive nitrogen oxide radical scavenging activity of (a) gallic acid and NbSe_2_ nanoparticles synthesized at (b) RT, (c) 70 °C, and (d) 100 °C.

The reactive nitrogen oxide (RNO) scavenging activity of NbSe_2_ nanoparticles synthesized at RT, 70 °C, and 100 °C was assessed, with gallic acid serving as the reference antioxidant. The results revealed negligible RNO scavenging potential for all nanoparticle samples, Fig. 16. The samples synthesized at 70 °C and 100 °C showed minimal activity, with IC_50_ values exceeding 1000 μg mL^−1^, indicating their inability to neutralize 50% of the RNO radicals even at the highest tested concentration. The RT nanoparticle sample exhibited no detectable scavenging activity, confirming its lack of interaction with RNO radicals at any tested concentration. Gallic acid demonstrated a highly effective RNO scavenging capacity, with an IC_50_ of 1.59 ± 0.07 μg mL^−1^. This stark difference underscores the weak antioxidant activity of the NbSe_2_ nanoparticles compared to the potent scavenging ability of the standard antioxidant. Bai *et al.* (2024) reported similar results, in which cerium oxide nanoparticles showed RNO scavenging capacity.^44^ These findings suggest that NbSe_2_ nanoparticles synthesized under the evaluated conditions lack significant RNO scavenging properties, limiting their utility in applications requiring strong antioxidant performance.

The antioxidant activity of NbSe_2_ nanoparticles demonstrated a clear dependence on the synthesis temperature, with distinct variations observed across the assays. The temperature-induced changes in the particle size, morphology, and surface properties are likely the key factors influencing their antioxidant potential. The nanoparticles synthesized at RT exhibited superior performance in the CUPRAC and hydroxyl radical scavenging assays. This suggests that lower synthesis temperatures preserve surface characteristics that enhance electron transfer and radical interactions, possibly due to a higher density of active sites or an improved surface charge distribution. The strong antioxidant activity of the RT sample in the hydroxyl radical scavenging assay and its ability to surpass the standard Trolox in the CUPRAC assay highlight the efficacy of this synthesis condition in producing highly active antioxidant nanoparticles.

The 70 °C sample showed the highest antioxidant activity in the FRAP assay, suggesting that moderate heating may optimize certain particle properties, such as crystalline structure and particle dispersion, which are favorable for iron-reducing activity. However, the reduced activity of the 70 °C sample in hydroxyl and CUPRAC assays indicates that the synthesis temperature may alter specific surface characteristics critical for interacting with radicals other than iron. In contrast, nanoparticles synthesized at 100 °C demonstrated significant activity only in the superoxide anion scavenging assay, comparable to the standard gallic acid. This finding suggests that higher synthesis temperatures induce structural or compositional changes, such as increased crystallinity or surface oxidation, which selectively enhance interactions with superoxide radicals. However, the diminished performance of the 100 °C sample in other assays of CUPRAC and hydroxyl radical scavenging indicates a trade-off between thermal stability and the availability of functional sites for broader antioxidant activity.

Most researchers have used silver nanoparticles and their variants to assess antioxidant potential.^45,46^ Netala *et al.* (2016) reported that Ag nanoparticles exhibited significant DPPH radical scavenging activity, with an IC_50_ value of 60.64 μg m^−1^, indicating that metal nanoparticles have potential as antioxidant agents.^47^ Chandra *et al.* (2019)^65^ reported ZnO nanoparticles showing DPPH radical scavenging activity with an IC_50_ value of 3.55 μg mL^−1^. Sathiyaseelan (2020) reported that silver nanoparticles showed noticeable ABTS, DPPH, hydroxyl radical, and superoxide radical scavenging activity, which is on par with the outcomes of the present study that indicate a wide spectrum of antioxidant potential of metal nanoparticles.^48^ There have been many reports of metal nanoparticles displaying antioxidant activities that can be potentially beneficial in oxidation damage and ROS-induced disorders and diseases such as cancer, Alzheimer's disease, and fibrosis (Ferreira *et al.*, 2018; Morry *et al.*, 2017).^49,50^ Although the exact underlying mechanism needs to be investigated, as per Samrot *et al.* (2022), the multi-ionization state and reactivity of the metals are the primary reasons they can act as excellent antioxidant agents.^51^

The antioxidant performance of NbSe_2_ nanoparticles is closely linked to their synthesis temperature, which governs their particle size, morphology, and surface chemistry. Our results indicate that 70 °C provides the most favorable balance, producing moderately crystalline, layered particles with adequate surface activity. Optimizing the temperature, growth conditions, and potential post-synthesis modifications can significantly enhance the antioxidant functionality by fine-tuning surface defects and active sites.

#### 3.5.3. *In vitro* cytotoxicity evaluation of NbSe_2_ nanoparticles

**Fig. 17:**
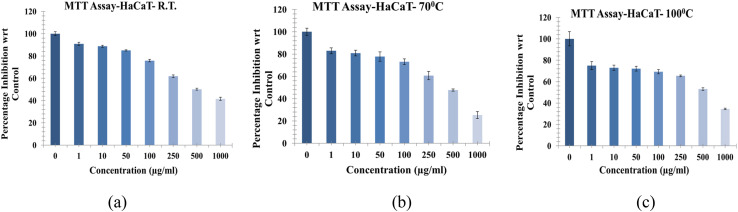
*In vitro* cytotoxicity of NbSe_2_ nanoparticles synthesized at (a) RT, (b) 70 °C, and (c) 100 °C.

The cytotoxic activity of NbSe_2_ nanoparticles synthesized at RT, 70 °C, and 100 °C was evaluated using the HaCaT cell line (human keratinocytes). The sample synthesized at 70 °C exhibited the highest cytotoxic activity, with an IC_50_ of 360.91 ± 0.15 μg mL^−1^, indicating its stronger inhibitory effect on cell proliferation, Fig. 17. The RT sample showed moderate cytotoxicity, with an IC_50_ of 568.80 ± 0.07 μg mL^−1^, requiring higher concentrations to achieve similar cell growth inhibition. The 100 °C sample displayed the lowest cytotoxic activity, with an IC_50_ of 744.90 ± 0.37 μg mL^−1^, indicating a comparatively weaker effect on HaCaT cells. These results confirm that the synthesis temperature can affect the cytotoxicity of NbSe_2_ nanoparticles. The higher cytotoxicity observed for the 70 °C sample may result from changes in the particle size or surface properties or reduced aggregation, which enhance interactions with cellular membranes. Conversely, the reduced activity in the case of the higher synthesis temperature of 100 °C suggests that altered surface or structural features reduce bioactivity. While the nanoparticles demonstrate cytotoxicity, their IC_50_ values are relatively high compared to standard cytotoxic agents, indicating moderate levels of cytotoxicity only at high concentrations. Heavy metals are known to show cytotoxicity; however, their lower concentration and derivatives have been used for medicinal purposes. Pathak *et al.* (2019) reported *in vitro* cytotoxicity against HepG-2 and MCF-7 breast cancer cell lines using silver nanoparticles at concentrations of 62.5 and 125 μg mL^−1^, respectively, which is significantly lower than what is reported in the present study, which indicates that NbSe_2_ nanoparticles have lower cytotoxicity than previously reported metal nanoparticles.^52^ There have been studies on the assessment of the cytotoxicity of metal nanoparticles ranging from silver, gold, zinc, and iron to tin.^53–56^ Dinesh *et al.* (2017) reported the cytotoxicity of Pd nanoparticles at a 150 μg mL^−1^ concentration against HeLa cell lines.^57^ Maheswari *et al.* (2021) reported the cytotoxicity of TiO_2_ nanoparticles at a meagre 50 μg mL^−1^ concentration against KB oral cancer cell lines.^58^ Although NbSe_2_ nanoparticles have shown low cytotoxicity in short-term *in vitro* studies, further investigations are required to assess their safety under prolonged exposure or higher doses. Future studies should include long-term, dose-dependent biological evaluations using oxidative stress markers and apoptotic pathways to ensure comprehensive biocompatibility for clinical applications.

#### 3.5.4. Wound healing potential of NbSe_2_ nanoparticles

**Fig. 18:**
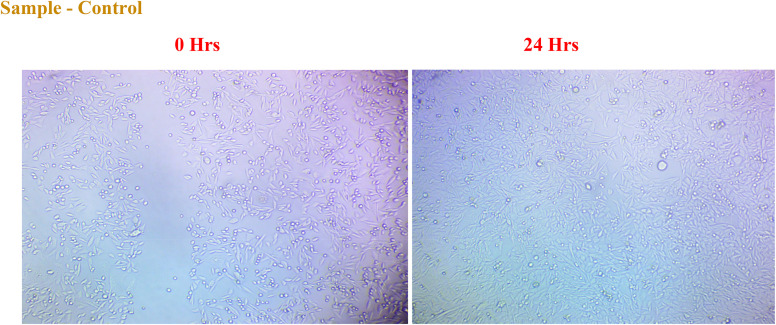
*In vitro* wound healing scratch assay images of the control at 0 h and 24 h.

**Fig. 19:**
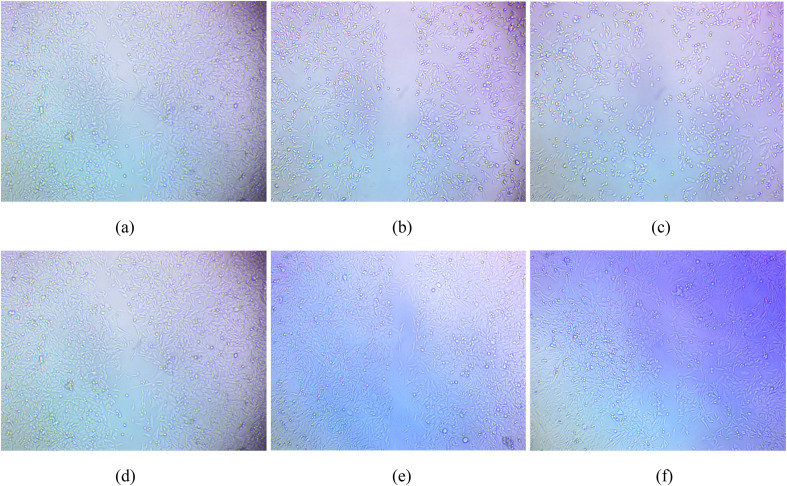
*In vitro* wound healing scratch assay images at 0 h (a–c) and 24 h (d–f) of NbSe_2_ nanoparticles synthesized at (a and d) RT, (b and e) 70 °C, and (c and f) 100 °C for different dosages of 0.5, 1, and 2 μg mL^−1^.

The *in vitro* scratch assay was used to evaluate the wound healing potential of NbSe_2_ nanoparticles synthesized at RT, 70 °C, and 100 °C. The assay measured cell migration and wound closure, with recovery percentages assessed at specific doses and time points. Fig. 18 shows the *in vitro* wound healing scratch assay images of the control at 0 h and 24 h while Fig. 19 shows the *in vitro* wound healing scratch assay images of NbSe_2_ nanoparticles synthesis at (a) RT, (b) 70 °C, and (c) 100 °C after 0 h and particles synthesized at (c) RT, (d) 70 °C, and (e) 100 °C for 24 h at different dosages of 0.5, 1, and 2 μg mL^−1^.

**Fig. 20:**
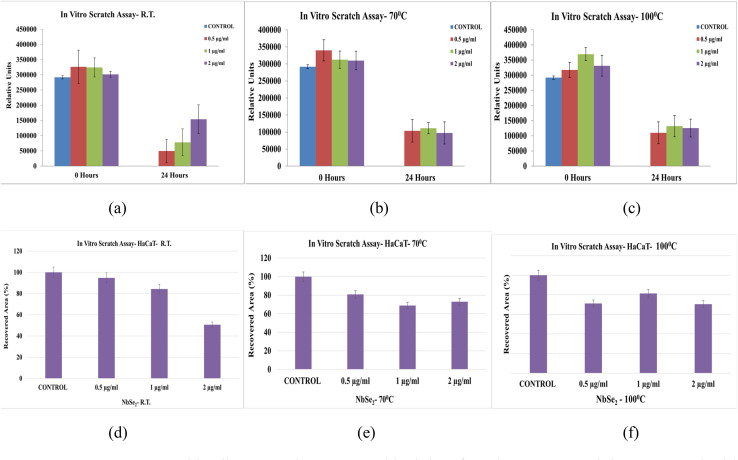
*In vitro* wound healing scratch assay graphical data for NbSe_2_ nanoparticles as the control with 0.5 μg per mL dosages that are synthesized at (a) RT, (b) 70 °C, and (c) 100 °C and for dosage of 1 and 2 μg mL^−1^ for the synthesis temperatures of (d) RT, (e) 70 °C, and (f) 100 °C.

The RT sample exhibited the highest efficacy, achieving 94.78% wound closure within 24 hours at 0.5 μg mL^−1^, Fig. 20(a and d). The 70 °C sample, Fig. 20(b and e), showed 80.89% recovery under the same conditions, while the 100 °C sample, Fig. 20(c and f), achieved 81.39% recovery but required a higher dose of μg mL^−1^. The wound healing promotion ability of the metal nanoparticles has been well documented before by Wang *et al.* 2024.^59^

Aydin Acar *et al.* (2024) reported ZnO nanoparticles showing partial cell migration and percent wound closure of 69.1% in L929 cells, a mouse fibroblast cell line.^60^ The present study showed a high level of wound healing capability compared to previous similar studies. Copper, silver, and zinc nanoparticles have been reported for their potential to assist in wound healing.^61^ According to Zheng *et al.* (2024), the wound healing properties of the metal nanoparticles can be attributed to their anti-microbial property, anti-inflammatory response, and promotion of angiogenesis.^62^

The findings of this study demonstrate that the synthesis temperature plays a critical role in determining the wound-healing efficiency of NbSe_2_ nanoparticles, likely due to the associated changes in the particle size, surface characteristics, and biological interactions. Among the samples tested, the room temperature (RT) synthesized nanoparticles exhibited the most promising wound-healing performance. Further investigations are required to optimize synthesis parameters and elucidate the underlying mechanisms driving their therapeutic effects for potential applications in regenerative medicine. Moreover, owing to their unique physicochemical properties, NbSe_2_ nanoparticles hold significant potential for future development as theranostic agents capable of integrating multimodal imaging with antibacterial and wound-healing functionalities.

## 4. Conclusions

The NbSe_2_ nanoparticles synthesized at RT, 70 °C, and 100 °C exhibited distinct structural, morphological, and biological properties, demonstrating the importance of synthesis conditions in tailoring their performance for potential biomedical applications. The XRD analysis confirmed the successful synthesis of the NbSe_2_ phase with a hexagonal structure, with slight variations in crystallinity observed with temperature changes. The SEM and TEM analyses revealed that higher synthesis temperatures led to slightly larger nanoparticles with improved dispersion, as seen in the cases of 70 °C and 100 °C samples compared to the RT sample. The EDX confirmed the stoichiometry of all three different-temperature synthesized NbSe_2_ nanoparticles.

Biologically, the cytotoxicity assays showed that the nanoparticles synthesized at 70 °C exhibited the highest cytotoxicity (IC_50_ of 360.90 ± 0.15 μg mL^−1^), while the RT sample exhibited moderate cytotoxicity (IC_50_ of 568.80 ± 0.07 μg mL^−1^), and the 100 °C sample displayed the lowest cytotoxicity (IC_50_ of 744.90 ± 0.37 μg mL^−1^). In antimicrobial testing, the RT sample demonstrated the highest inhibition against *Aspergillus niger* and *Candida albicans*, suggesting that synthesis temperature influences antimicrobial activity. The wound healing assays revealed that the RT synthesized NbSe_2_ nanoparticles exhibited the highest wound closure potential (94.78% recovery at 0.5 μg mL^−1^), highlighting their promising application in regenerative medicine. These findings underscore the potential of NbSe_2_ nanoparticles as bioactive materials, where their synthesis temperature plays a critical role in modulating their biological activity for medical applications. Given their unique physicochemical properties, NbSe_2_ nanoparticles also hold promise for future development as theranostic agents integrating multimodal imaging with antibacterial and wound-healing functions.

## Author contributions

SRB contributed to the material preparation, data collection, analysis, and writing of the original draft. MSD acquired resources and assisted in project administration, reviewing, and editing of the manuscript, and supervision. SHC participated in the conceptualization, investigation, and review of the manuscript. TAL participated in the conceptualization, investigation, data analysis, and review. ACK and AB participated in the biological methodology, writing, and data analysis of the manuscript.

## Conflicts of interest

There are no conflicts to disclose.

## Data Availability

The datasets generated/analyzed during the current study are presented in the article.
